# VEGF-D: a novel biomarker for detection of COVID-19 progression

**DOI:** 10.1186/s13054-020-03079-y

**Published:** 2020-06-23

**Authors:** Yaxian Kong, Junyan Han, Xueying Wu, Hui Zeng, Jingyuan Liu, Henghui Zhang

**Affiliations:** 1 Beijing Key Laboratory of Emerging Infectious Diseases, Institute of Infectious Diseases, Beijing Ditan Hospital, Capital Medical University, Beijing, 100015 China; 2 Immupeutics Medicine Institute, Beijing, 100191 China; 3 Intensive Care Medicine, Beijing Ditan Hospital, Capital Medical University, Beijing, 100015 China

As the coronavirus 2019 (COVID-19) continues to spread globally, hundreds of thousands have been infected, among whom approximately 15% of COVID-19 patients develop severe disease, and 5 to 6% are critically ill [1]. Critical patients of COVID-19 have a dramatically higher case fatality rate than severe cases. Thus, it is increasingly urgent to develop early and effective predictors to distinguish critical patients from severe patients. Storms of inflammatory cytokines and blood clots were reported to associate with severe disease and fatality of COVID-19 patients [2, 3]. We aimed to identify a biomarker for the detection of COVID-19 progression from numerous cytokines and coagulation indicators.

We conducted a retrospective study based on patients with a laboratory-confirmed diagnosis of COVID-19 admitted to the intensive care unit in Beijing Ditan Hospital from January 20, 2020, to March 23, 2020. This study was approved by the Ethics Committee of Beijing Ditan Hospital. The severity of COVID-19 was defined according to the guidelines on the diagnosis and treatment of new coronavirus pneumonia (version 7). All baseline medical record information including demographic, data, complications, and laboratory results were obtained within the first day after hospital admission. Blood samples were collected at baseline and once every 3–7 days during hospitalization. Forty-five cytokines/chemokines/growth factors in serum were measured using Luminex multiplex assay. Random forests machine learning classifier in Python environment was used for variable importance of the feature rankings. A receiver operating characteristic (ROC) curve was generated to evaluate the diagnostic accuracy of a protein.

A total of 24 COVID-19 patients were enrolled in this study, including 14 (58.3%) severe patients and 10 (41.7%) critical patients (Table 1). Compared to the severe group, critical cases were of significantly older ages and showed higher white blood cell counts and neutrophil counts. Levels of VEGF-D, TNF-α, SCF, LIF, IL-2, IL-4, IL-6, IL-8, IL-10, IL-15, IL-17A, IL-18, IL-1β, and IFN-γ were significantly higher in the critical group than in the severe group (Table 1). Additionally, lymphocyte count, CRP, LDH, and coagulation indicators (d-dimer, platelet count, PT, and APTT), which were reported to associate with clinical outcome [4, 5], were also included in the random forests model.

**Table 1:** Demographics, baseline characteristics, cytokines, chemokines, and growth factors of COVID-19 patients

Characteristics	Total (*n* = 24)	Severe patients (*n* = 14)	Critical patients (*n* = 10)	*P* values
Age mean range, years	68 (36, 88)	65 (36, 81)	77 (64, 88)	**.003**
Gender				.521
Male, *n* (%)	15 (62.5)	10 (71.4)	5 (50)	
Female, *n* (%)	9 (37.5)	4 (28.6)	5 (50)	
Admission to ICU, mean (SD), days	22 (22)	13 (8)	35 (28)	**.009**
SOFA score, mean (SD)	3.7 (2.4)	2.8 (1.7)	4.9 (2.8)	**.015**
Complications, *n* (%)				.568
Hypertension	10 (41.7)	6 (42.9)	4 (40.0)	1
Cardiovascular disease	4 (16.7)	1 (7.1)	3 (30.0)	.355
Chronic Pulmonary disease	6 (25)	1 (7.1)	5 (50.0)	.056
Diabetes	6 (25)	2 (14.3)	4 (40.0)	.339
Hyperlipemia	0	0	0	
Chronic kidney disease	3 (12.5)	2 (14.3)	1 (10.0)	1
Immune disorders	3 (12.5)	3 (21.4)	0	.348
Others	1 (4.2)	1 (7.1)	0	1
Laboratory data, mean (SD)				
WBC, 10^9^/L	7.72 (5.12)	5.82 (2.13)	10.20 (6.8)	**.039**
Lymphocyte, 10^9^/L	1.08 (0.47)	0.93 (0.45)	1.27 (0.44)	.089
Neutrophil, 10^9^/L	6.34 (4.91)	4.59 (1.74)	8.63 (6.7)	**.048**
Platelets, 10^9^/L	211 (98)	206 (85)	218 (117)	.785
PT, s	13.1 (1.7)	13.5 (1.9)	12.7 (1.4)	.291
APTT, s	33.8 (8.6)	33.9 (7.2)	33.7 (10.4)	.973
d-dimer, mg/L	4.9 (7.6)	2.9 (5.2)	7.1 (9.3)	.213
CRP, mg/L	69.4 (66.1)	55.3 (32.7)	85.1 (89.6)	.315
LDH, U/L	403.6 (129.6)	398.3 (66.1)	406.3 (155.5)	.916
Serum creatinine, μmol/L	108.1 (172.4)	67.9 (12.9)	148.1 (242.9)	.331
ALT, U/L	40.5 (13.9)	44.8 (23.2)	41.9 (31.5)	.809
Blood potassium, mmol/L	4.0 (0.5)	3.9 (0.4)	4.2 (0.5)	.299
Blood sodium, mmol/L	137.4 (5.9)	137.1 (8.2)	137.8 (2.7)	.772
Cytokines, chemokines, and growth factors, median (IQR), pg/mL				
VEGF-D	40.1 (17.7, 64.8)	25.9 (12.3, 40.6)	62.9 (45.8, 79.6)	**.0048**
TNF-α	25.3 (3.2, 67.9)	8.6 (0, 48.4)	54.8 (15.3, 131.0)	**.027**
SCF	17.1 (9.2, 20.7)	13.9 (7.7, 18.4)	20.1 (16.2, 68.3)	**.019**
LIF	18.4 (4.2, 64.9)	7.4 (1.9, 21.9)	56.5 (10.8, 96.9)	**.0089**
IL-2	35.2 (8.7, 59.1)	17.5 (4.7, 43.5)	55.2 (23.5, 90.2)	**.018**
IL-4	2.1 (0, 20.2)	0 (0, 9.8)	143.7 (47.2, 203.9)	**.033**
IL-6	54.2 (26.7, 157.8)	35.4 (19.5, 76.9)	143.7 (47.2, 203.9)	**.019**
IL-8	20.1 (0.1, 44.2)	2.6 (0, 13.0)	13.0 (5.4, 17.8)	**.039**
IL-10	6.0 (1.4, 15.3)	3.9 (0.9, 6.2)	8.4 (3.3, 24.1)	**.038**
IL-15	20.1 (4.8, 44.2)	10.7 (1.8, 26.3)	38.7 (18.1, 83.3)	**.018**
IL-17A	19.8 (0.7, 55.3)	9.5 (0, 26.2)	50.0 (16.4, 109.4)	**.021**
IL-18	86.0 (19.8, 185.6)	29.2 (18.5, 109.1)	158.9 (91.8, 209.2)	**.046**
IL-1β	8.8 (2.1, 25.2)	4.4 (1.6, 15.8)	22.8 (7.8, 52.7)	**.022**
IFN-γ	17.6 (6.2, 29.9)	9.1 (3.6, 24.2)	26.4 (12.9, 53.2)	**.013**

*WBC* white blood cells, *CRP* C-reactive protein, *LDH* lactate dehydrogenase, *PT* prothrombin time, *APTT* activated partial thromboplastin time, *ALT* alanine aminotransferase, *VEGF* vascular endothelial growth factor; *TNF-α* tumor necrosis factor-alpha, *SCF* stem cell factor; *LIF* leukemia inhibitory factor, *IL* interleukin, *IFN* interferon

Strikingly, VEGF-D was identified as the most important indicator related to the severity of COVID-19 (ranked as 1, Fig. 1a). As expected, d-dimer, age, IL-6, and lymphocyte count associated with clinical outcomes of COVID-19 patients reported previously were also highly ranked. VEGF-D had a higher area under the curve (AUC) (0.836 (95% CI 0.648–1); Fig. 1b) than d-dimer (0.755 (95% CI 0.527–0.982); Fig. 1c). Consistently, VEGF-D levels were positively correlated with sequential organ failure assessment (SOFA) scores (Fig. 1d). As shown in Fig. 1e, critical patients had higher levels of VEGF-D than the severe cases during the whole course of hospitalization.

**Fig. 1:**
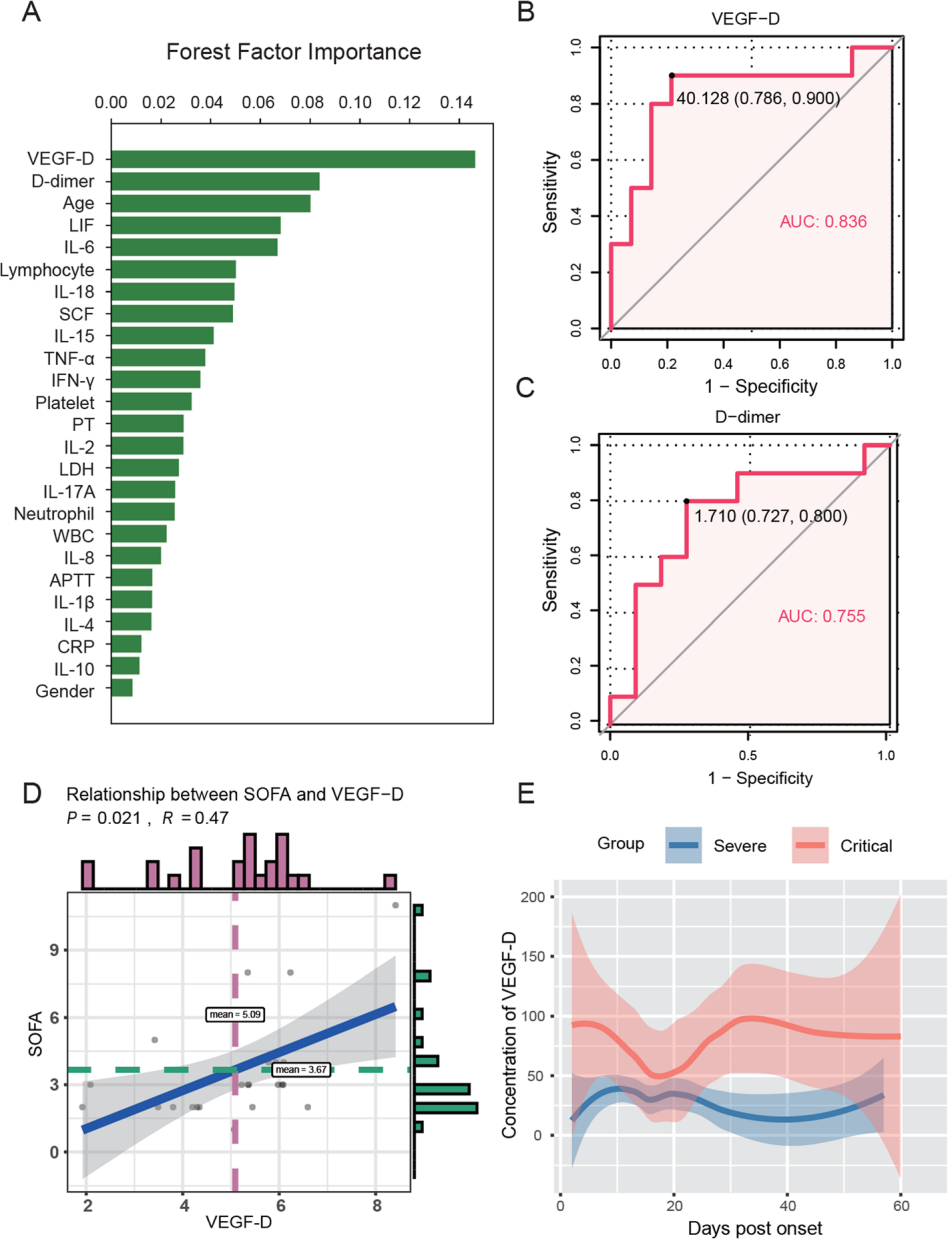
A high level of VEGF-D predicted progression of COVID-19. **a** Eleven clinical indicators and 14 cytokines were considered for inclusion and ranked by importance using random forest. **b**, **c** Receiver operating characteristic (ROC) analyses for VEGF-D (**b**) and d-dimer (**c**) in COVID-19 patients. **d** Relationship between VEGF-D and SOFA scores in severe and critical COVID-19 patients was analyzed by the Spearman rank correlation test. **e** Temporal changes of VEGF-D levels in each group during hospitalization. The median values of each time point (the day from onset) were shown. The 95% interval was plotted as a colored shadow

To our knowledge, this is the first report of VEGF-D as a potential biomarker for detecting the progression of COVID-19. Despite limited evidence in COVID-19, previous studies demonstrated an important role of VEGF in the pathogenesis of acute lung injury (ALI) and acute respiratory distress syndrome (ARDS) by its properties to increase vascular permeability. Furthermore, VEGF is regarded as an indirect procoagulant for altering the hemostatic features of endothelial cells [6]. We hypothesized that elevated VEGF-D level might potentially relate to the storm of blood clots occurring in COVID-19 patients. Notably, it is of great interest to investigate the therapeutic effects of VEGF inhibitor in COVID-19 patients.

This study has limitations, including the small sample size, a single-center experience, and a variable time interval of each patient from admission to symptoms onset. Studies based on a larger cohort in additional sites are needed to verify our findings.

## Data Availability

All data generated or analyzed during this study are included in this published article.
